# A case report of primary neuroendocrine carcinoma of the perihilar bile duct

**DOI:** 10.1186/s12893-015-0116-z

**Published:** 2015-12-10

**Authors:** Yasuhiro Kihara, Hiroshi Yokomizo, Takahiro Urata, Michiko Nagamine, Toshihiko Hirata

**Affiliations:** Division of General Surgery, Japanese Red Cross Kumamoto Hospital, Nagamineminami 2-1-1, Higashiku, Kumamoto city, Kumamoto 861-8520 Japan; Division of Gastroenterology, Japanese Red Cross Kumamoto Hospital, Kumamoto, Japan; Division of Diagnostic Pathology, Japanese Red Cross Kumamoto Hospital, Kumamoto, Japan

**Keywords:** Neuroendocrine carcinoma, Bile duct, Small cell carcinoma

## Abstract

**Background:**

Although neuroendocrine tumors are most commonly found in the digestive system, neuroendocrine tumors originating from the bile duct are rare, and neuroendocrine carcinomas derived from the perihilar bile duct are extremely rare. This report presents the clinical course and clinicopathological features of neuroendocrine carcinomas arising from the extrahepatic bile duct.

**Case presentation:**

A 70-year-old Japanese woman was preoperatively diagnosed with perihilar cholangiocarcinoma, and a radical resection with an extended left hepatic lobectomy and a choledochojejunostomy was performed. From the histopathological findings, we diagnosed the tumor as a neuroendocrine carcinoma of the bile duct (small cell type) with lymph node metastasis. The patient was treated with the same adjuvant chemotherapy as that used for small cell carcinoma of the lung. At 10 months after surgery, there was no recurrence of the disease.

**Conclusion:**

Neuroendocrine carcinoma of the extrahepatic biliary tracts is a very rare and highly malignant disease with a poor prognosis. A multidisciplinary approach could improve the prognosis for this neoplasm.

## Background

The World Health Organization (WHO) classification system (2010) defines neoplasms with neuroendocrine differentiation as neuroendocrine tumors (NETs). These are stratified as NET Grade 1 (G1), NET Grade 2 (G2), and neuroendocrine carcinoma (NEC) [1]. Although NETs are most commonly found in the digestive system, NETs originating from the extrahepatic bile duct are rare, and NECs arising from the perihilar bile duct are extremely rare [2]. In this report, we present the clinical course and clinicopathological features of a case of NEC originating from the extrahepatic bile duct, as well as the results of a literature review on this subject.

## Case presentation

**Fig. 1:**
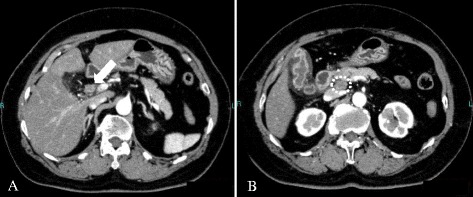
CT findings. Abdominal enhanced computed tomography showed an enhanced tumor located in the hilar bile duct to the left hepatic duct (**a**: *arrow*). The encircled area indicated the regional lymph node metastasis located along the common bile duct (**b**)

**Fig. 2:**
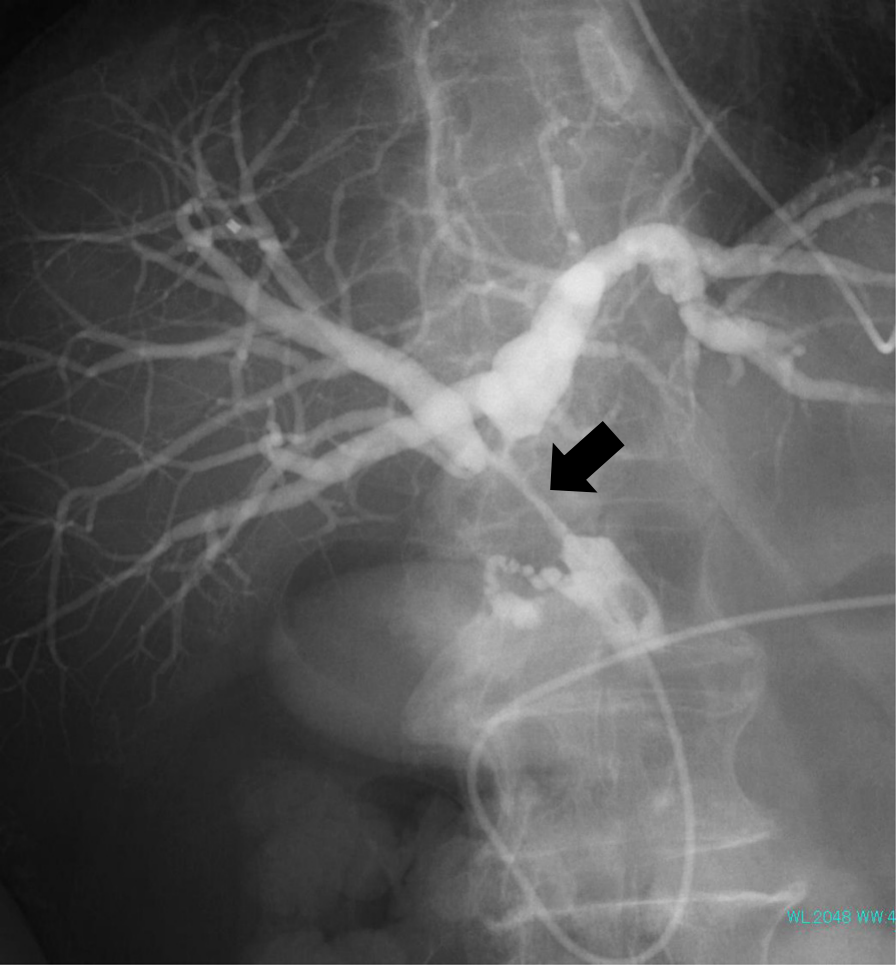
Cholangiography. Cholangiography revealed the obstruction of the hilar to upper portion of bile duct (*arrow*)

A 70-year-old Japanese woman presented to our hospital for investigation of a chief complaint of jaundice. The patient had no family history of cancer. A physical examination revealed no remarkable findings. Laboratory data showed abnormally elevated levels of total bilirubin (3.6 mg/dL; normal, 0.2-1.0 mg/dL), AST, ALT, and γ-GTP. The serum level of CEA was normal; however, the CA19-9 was above the normal range (47 U/ml; normal, 0–37 U/ml). An abdominal computed tomography (CT) scan revealed a 3 cm enhancing mass located in the hilar bile duct extending to the left hepatic duct (Fig. 1a) and an enlarged regional lymph node along the common bile duct (Fig. 1b). In recent years, for the preoperative biliary drainage in patients with malignant biliary tract obstruction, we prefer endoscopic retrograde drainage rather than percutaneous transhepatic drainage in regard to adverse events, such as vascular injury and cancer dissemination. Therefore, an endoscopic retrograde cholangiopancreatography (ERCP) was performed, and cholangiography revealed the obstruction of the hilar to upper portion of bile duct (Fig. 2). And endoscopic retrograde biliary drainage (ERBD) tube was placed. A bile duct brush cytologic specimen obtained at the time of ERCP showed small clusters of relatively small atypical cells with hyperchromatic nuclei and scant cytoplasm. A salt-and-pepper chromatin pattern and cell molding, which are typical in neuroendocrine tumors, were not apparent. The cytologic diagnosis at this point was adenocarcinoma, possibly poorly differentiated. The patient was diagnosed with hilar cholangiocarcinoma with regional lymph node involvement. Abdominal and chest CT scans showed no other neoplastic lesions. A radical resection was performed, including an extended left hepatic lobectomy, excision of the caudate lobe and the extrahepatic bile duct, dissection of the regional lymph nodes, and a choledochojejunostomy.

**Fig. 3:**
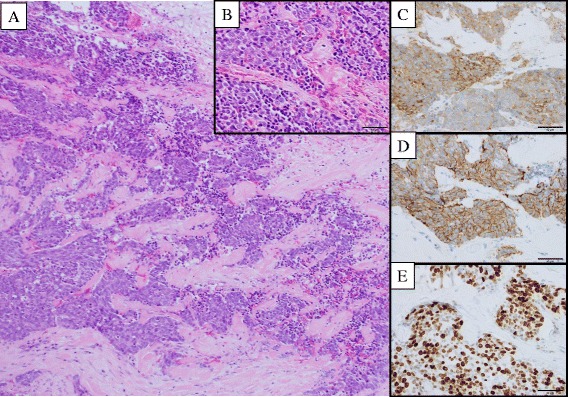
Pathological findings. Hematoxylin-eosin stained histologic sections of the resected perihilar bile duct. The tumor cells were arranged in the cellular nests and cords infiltrating the bile duct wall. The mucosa epithelium was mostly eroded (*upper right*) (**a**: ×100). The tumor cells were round or oval with scant cytoplasm. Numerous mitotic figures and apoptotic bodies are noted (**b**: ×400). In the immunohistochemical studies, the tumor cells were diffusely positive for synaptophysin (**c**: ×400), and CD56 (**d**: ×400). Approximately 70 % of the tumor cells were positive for Ki-67 (**e**: ×400)

In the resected specimen, the mucosa of the perihilar bile duct was diffusely rough, and the duct wall was thickened in a 3 cm portion of the proximal common hepatic duct. Microscopically, the thickened bile duct wall was infiltrated by irregular islands and nests of cells with hyperchromatic nuclei and a high nuclear-to- cytoplasmic ratio (Fig. 3a). The tumor cells were relatively small, approximately 2 to 3 times larger than the background lymphocytes (Fig. 3b). Rosette-like structures were not seen. Numerous mitotic figures and apoptotic bodies were present. Immunohistochemical stains showed the tumor cells to be positive for synaptophysin (Fig. 3c) and CD56 (Fig. 3d), and focally positive for chromogranin A. The Ki-67/MIB-1 Labeling Index was 70 % (Fig. 3e). Based on these findings, a pathological diagnosis of small cell neuroendocrine carcinoma was established. Two out of 8 regional lymph nodes were positive for metastatic carcinoma.

There were no postoperative complications, and the patient received 4 courses of adjuvant chemotherapy with a regimen of irinotecan (50 mg/m^2^) and carboplatin (5 AUC/body). Ten months after surgery, there was no recurrence of the disease.

## Discussion

NECs were previously classified as small cell carcinomas (SCCs), large cell neuroendocrine carcinomas, or poorly differentiated neuroendocrine carcinomas [3]. NEC is a poorly differentiated, high-grade, malignant neoplasm composed of small cells or intermediate to large cells. In the digestive system, NECs have been reported in the esophagus, the ampulla of Vater [4], the pancreas [5], and the gallbladder [6]. However, NECs in the bile duct are extremely rare.

We conducted a PubMed systematic literature search (1985–2014) using keywords such as “neuroendocrine carcinoma,” “small cell,” and “biliary tract,” and found only 23 reported cases of NEC of the extrahepatic biliary tracts, excluding the intrahepatic bile duct, the gallbladder, and the ampulla of Vater (Table 1, [7–29]). SCC was the most common histologic subtype of NEC of the extrahepatic bile ducts (19 of 23 cases; Table 1). There were only 3 cases of large cell carcinomas of the common bile duct (cases 21 to 23, Table 1). NEC can occur anywhere in the extrahepatic bile duct, but the middle portion of the common bile duct appears to be the most common site of involvement. The prognosis for NEC of the bile duct appears to be poor. Of the 23 cases with follow-up data, 57 % (12/23) of the patients died 3 to 20 months after surgery, and only 2 patients were reported to have survived more than 2 years. NEC of the biliary system has a high incidence of distant metastasis [15, 16, 19, 20, 22, 24–29]. Consequently, it has a poor prognosis, and surgical resection alone is not an effective treatment. A report by Levenson revealed that there is no survival benefit from using surgery to treat either small cell lung cancer or extrapulmonary SCC [30]. This may be because the most important prognostic factor is the extent of disease at diagnosis, and most patients with extrapulmonary SCC already have occult metastasis [30]. Of the 12 patients died within 20 months after surgery, 9 cases were identified their recurrence pattern and all of them were distant metastases [15, 16, 19, 20, 22, 24, 26, 28, 29]. Meanwhile, the long survival 2 cases had locoregional lymph node metastases without distant metastasis [13, 27]. Since NEC of the biliary system had a high incidence of distant metastasis, locolegional lymph node metastasis could not be a prognostic factor. In the report of 37 cases of neuroendocrine tumor of ampulla of Vater, the authors did not find any prognostic value of the locoregional lymph node metastases and lymphadenectomy [31].

**Table 1:** Neuroendocrine carcinoma of the extrahepatic bile duct. Review of the literature

No.	Author	Age	Sex	Histology	Location	Size	Treatment	Prognosis
1	Sabanathan (1988)	67	M	Small cell	Bm	5 cm	Palliative bypass and chemo.	6 months, alive
2	Van der Wal (1990)	55	M	Small cell + Adenoca.	Bm	4 cm	Resection	N.A.
3	Nishihara (1993)	64	M	Small cell + Adenoca.	Bh-Bs	1.9 cm	Resection	8 months, alive
4	Yamamoto (1998)	71	F	Small cell + Adenoca.	Bh	6 cm	Resection	8 months, dead
5	Kim (2000)	64	M	Small cell + Adenoca.	Bm	3 cm	Resection	1 month, alive
6	Miyashita (2001)	85	F	Small cell	Bi	3 cm	Palliative bypass	5 months, dead
7	Edakuni (2001)	82	F	Small cell + Adenoca.	Bm	6 cm	Resection	45 months, alive
8	Kuraoka (2003)	75	M	Small cell	Bi	4.5 cm	Resection	5 months, alive
9	Hazama (2003)	60	M	Small cell	CBD	0.3 cm	NAC and resection	12 months, dead
10	Arakura (2003)	70	F	Small cell	Bm	3 cm	Resection and chemo.	14 months, dead
11	Park (2004)	60	F	Small cell	Bs-Bm	3 cm	Resection	5 months, dead
12	Thomas (2005)	54	M	Small cell	Bh-CBD	N.A.	Resection	6 months, alive
13	Kaiho (2005)	66	F	Small cell + Adenoca.	Bm	3.5 cm	Resection and chemo.	8 months, dead
14	Sato (2006)	68	M	Large cell + Adenoca.	Bi	2 cm	Resection and chemo.	3 months, dead
15	Viana Miguel (2006)	76	M	Small cell	Bm	N.A.	Resection, chemo. and irraiation	5 months, alive
16	Jeon (2006)	65	M	Small cell	Bs-Bm	2 cm	Resection and chemo.	12 months, dead
17	Nakai (2008)	32	M	Small cell	CBD	N.A.	N.A.	N.A.(autopsy)
18	Arakura (2008)	75	M	Small cell	Bh-Bs	6.5 cm	Chemo. and irradiation	10 months, dead
19	Hosonuma (2008)	69	F	Small cell	Bs-Bm	3 cm	Biliary drainage	2 months, alive
20	Okamura (2009)	62	M	Small cell	Bm	3 cm	NAC, resection and irradiation	20 months, dead
21	Yamaguchi (2009)	77	F	NEC	Bi	N.A.	Resection and chemo.	27 months, alive
22	Demoreuil (2009)	73	M	Large cell + Adenoca.	Bh-Bs	3 cm	Resection and chemo.	12 months, dead
23	Sasatomi (2013)	76	M	Large cell	Bh-Bs	5 cm	Resection	21 days, dead
24	Current report (2014)	70	F	Small cell	Bh	5 cm	Resection and chemo.	10 months, alive

*NEC* neuroendocrine carcinoma, *NAC* neo adjuvant chemotherapy, *Adenoca*. adenocarcinoma, *CBD* common bile duct, *Bh* hilar bile duct, *Bs* superior portion of common bile duct, *Bm* mid portion of bile duct, *Bi* inferior portion of bile duct, *chemo.* chemotherapy, *N.A.* not available, *M* male, *F* female

If there is a biopsy-proven preoperative diagnosis of NEC, then preoperative chemotherapy can improve the prognosis in comparison to surgery alone or surgery with adjuvant chemotherapy. Hazama et al. revealed that neoadjuvant chemotherapy followed by surgery resulted in an excellent response for SCC of the common bile duct [15]. Okamura et al. reported that multidisciplinary management, consisting of preoperative chemotherapy, a curative resection, adjuvant chemotherapy, and radiation therapy, may scontribute to a prolonged survival for SCC of the common bile duct [26]. In most cases of NEC of the biliary system diagnosed from pathological findings of resected specimens, surgical resection followed by adjuvant chemotherapy is the generally accepted optimal treatment.

Although there is no established standard treatment for extrapulmonary SCC, chemotherapy should be attempted, if possible, because SCC is often chemosensitive [32]. The recommended chemotherapy regimen for extrapulmonary SCC is the same as that for small cell lung cancer. For patients with a diagnosis of the small cell type of NEC who are able to undergo surgical resection, adjuvant chemotherapy consisting of cisplatin and etoposide is also recommended for prevention of systemic recurrence [33, 34].

## Conclusion

In summary, neuroendocrine carcinoma of the extrahepatic biliary tracts is a very rare and highly malignantdisease with a poor prognosis. Although treatment strategies have not yet been established, a multidisciplinary approach at diagnosis of NEC of the biliary system, as well as further studies on therapeutic management could improve the prognosis of this highly malignant neoplasm.

### Consent

A written informed consent was obtained from the patient for the publication of this case report, along with all corresponding figures. A copy of the consent is available for review by the editors of this journal.

## Abbreviations

CA19-9: carbohydrate antigen 19–9
CEA: carcinoembryonic antigen
ERCP: endoscopic retrograde cholangiopancreatography
NEC: neuroendocrine carcinoma
NET: neuroendocrine tumour
SCC: small cell carcinoma
